# Early changes in cardiac troponin T and NT-proBNP levels in neonates receiving ECMO support: a single-center experience

**DOI:** 10.1186/s12872-024-03899-8

**Published:** 2024-04-30

**Authors:** Wen-Peng Xie, Yi-Nan Liu, Ya-Ting Zeng, Yi-Rong Zheng, Qiang Chen

**Affiliations:** Department of Cardiac Surgery, Fujian Children’s Hospital (Fujian Branch of Shanghai Children’s Medical Center), College of Clinical Medicine for Obstetrics & Gynecology and Pediatrics, Fujian Medical University, Fuzhou, China

**Keywords:** Neonates, ECMO, cTnT, NT-proBNP early results

## Abstract

**Objective:**

This study aimed to examine the changes in absolute value and decline rate of early serum cardiac troponin T (cTnT) and N-terminal pro b-type natriuretic peptide (NT-proBNP) in neonates who received veno-arterial (V-A) extracorporeal membrane oxygenation (ECMO) support therapy within the first week of life.

**Methods:**

We retrospectively collected clinical data and laboratory test results of 18 neonates who underwent V-A ECMO support within one week of birth, from July 2021 to June 2023, using the electronic medical record system. These patients were categorized into survival and death groups. Comparative analyses of the absolute values and decline rates of cTnT and NT-proBNP were made between the groups at baseline, and at 24, 48, and 72 h post-ECMO initiation.

**Results:**

Out of the 18 neonates, 12 survived (survival rate: 66.7%), while 6 succumbed. The survival group exhibited significantly lower absolute values of cTnT and NT-proBNP than the death group, and their decline rates were significantly higher. Notably, all neonates without an early decline in cTnT and NT-proBNP levels were in the death group.

**Conclusion:**

The early changes in the absolute value and decline rate of serum cTnT and NT-proBNP in neonates undergoing V-A ECMO may serve as predictors of their prognosis.

## Background

Neonatal respiratory and circulatory failure is a leading cause of critical illness and neonatal mortality [1, 2]. With advancements in respiratory support technologies such as pulmonary surfactant (PS), high-frequency oscillatory ventilation (HFOV), and inhaled nitric oxide (iNO), many neonates receive effective treatment. However, severe respiratory failure in newborns often necessitates extracorporeal membrane oxygenation (ECMO) support. Currently, ECMO is widely implemented in advanced neonatal intensive care units (NICU) for conditions like severe meconium aspiration syndrome (MAS), acute respiratory distress syndrome (ARDS), congenital diaphragmatic hernia (CDH), persistent pulmonary hypertension of the newborn (PPHN), and severe lung infections, with an overall survival rate ranging from 51 to 95% [1, 2]. Cardiac troponin T (cTnT) is a specific marker for myocardial cell injury, extensively used in clinical settings with high predictive value for various diseases causing primary and secondary myocardial damage [3, 4]. Similarly, the increase of N-terminal pro-brain natriuretic peptide (NT-proBNP) is highly valuable in assessing myocardial function and disease progression in cardiovascular patients [5, 6]. There are limited reports on the early changes of cTnT and NT-proBNP in low-weight neonates within one week of birth receiving veno-arterial (V-A) ECMO. We hypothesize that early elevated levels of cTnT/NT-proBNP are associated with a poorer prognosis in neonates on V-A ECMO. Therefore, this study aims to explore this hypothesis based on our clinical experience, providing insights for early treatment strategies in these patients.

## Methods and material

### Patient

This retrospective study received approval from our hospital’s ethics committee. We selected neonatal patients who received V-A ECMO treatment between July 2021 and June 2023 from the electronic medical record system. We exclusively utilized the V-A ECMO treatment model. Their clinical data and laboratory test results were collected for analysis. The exclusion criteria were as follows: 1) age greater than seven days at the time of ECMO initiation; 2) incomplete medical data; 3) refusal of participation by family members; 4) after cardiac surgery. Based on these inclusion and exclusion criteria, 18 neonatal patients were ultimately included in the study. They were divided into two groups according to their survival status: a survival group (*n* = 12) and a death group (*n* = 6).

### ECMO indications and contraindications

In this study, the indications for ECMO included: an oxygenation index (OI) greater than 40 for over 4 h; OI exceeding 20 for more than 24 h, or persistently worsening dyspnea (calculated as mean airway pressure (MAP) × fraction of inhaled oxygen (FiO_2_) × 100 / arterial partial pressure of oxygen (PaO_2_)); continued deterioration with severe hypoxemia (PaO_2_ < 40 mmHg) despite active respiratory support; arterial blood gas analysis indicating pH < 7.15, blood lactic acid ≥ 5 mmol/L, and urine output < 0.5 ml/(kg∙h) for 12–24 h; and pulmonary hypertension leading to right ventricular dysfunction, requiring continuous high-dose inotropic drugs for cardiac function maintenance (vasoactive inotropic score (VIS) > 40 points), with VIS calculated as dopamine (μg/kg/min) + dobutamine (μg/kg/min) + 10 × milrinone (μg/kg/min) + 100 × epinephrine (μg/kg/min) + 100 × norepinephrine (μg/kg/min) + 10000 × pituitrin (units/kg/min) [7]. Contraindications for ECMO included fatal congenital abnormalities, grade III or higher intraventricular hemorrhage, uncontrolled bleeding, and other irreversible brain injuries [8].

### ECMO strategy

The ECMO system comprises a MAQUET centrifugal pump, a MEDOS 800LT membrane oxygenator, an air-oxygen mixer, a circulation line, and a heating system. Veno-arterial cannulation, sedation, anesthesia, and muscle relaxation were implemented in all cases. Cannulation involved the right internal jugular vein (10 Fr) and the right common carotid artery (8 Fr), adhering to surgical guidelines and sterile techniques. Before cannulation, heparin (1 mg/kg) was administered. The circuit was primed with 200 ml of Ringer’s solution and 10 mg of heparin to eliminate air, followed by the addition of albumin and suspended red blood cells to replace the crystalloid in the circuit. Subsequently, 5% sodium bicarbonate (5–10 ml) and 10% calcium gluconate (3 ml) were added. The ECMO weaning process entailed reducing the flow rate to 50 ml/kg/min, adjusting ventilator settings to standard parameters, and monitoring for 3–6 h. Arterial blood gas analysis was utilized to assess lung ventilation and systemic oxygenation. Once vital signs stabilized, ECMO was discontinued, and arterial and venous cannulas were removed.

### Measurement of cTnT and NT-proBNP

Our center routinely assessed the levels of cTnT and NT-proBNP in patients’ venous blood at the initiation of ECMO therapy, and subsequently at 24, 48, and 72 h post-ECMO initiation. The decline rate of cTnT was calculated as follows: (initial cTnT – cTnT at 24 h, 48 h, or 72 h) / initial cTnT. Similarly, the decline rate of NT-proBNP was determined by: (initial NT-proBNP – NT-proBNP at 24 h, 48 h, or 72 h) / initial NT-proBNP.

### Pulmonary artery pressure

Echocardiography, valued for its noninvasive nature and ease of use, is widely adopted in clinical settings for estimating pulmonary artery pressure in infants [9]. Doppler echocardiography offers a noninvasive means to evaluate pulmonary artery pressure through the peak velocity of tricuspid regurgitation (TR Vmax) along with its associated parameters. These include the TR pressure gradient (TR-PG), the TR mean pressure gradient (TR-mPG), the estimated mean pulmonary artery pressure (mPAP), and the systolic pulmonary artery pressure (sPAP). The TR-PG is determined from the TR Vmax captured via continuous-wave Doppler using the simplified Bernoulli equation: TR-PG = 4 × (TR Vmax) [2]. The sPAP is then derived by adding the estimated right atrial pressure (RAP) to TR-PG [10]. In this study, an sPAP > 30 mmHg served as the echocardiographic threshold for diagnosing pulmonary arterial hypertension (PAH), with sPAP levels between 30 and 45 mmHg classified as mild, those between 45 and 70 mmHg as moderate, and sPAP > 70 mmHg indicating severe PAH [11].

### Definition of PPHN and ARDS

PPHN, formerly known as persistent fetal circulation, is characterized by failed circulatory transition at birth. PPHN’s hallmark physiological features include sustained pulmonary vascular resistance and ongoing hypoxemia post-delivery [12]. Currently, PPHN remains a leading cause of critical conditions in the NICU. ARDS is identified as an acute inflammatory lung condition, arising from various causes, that leads to pathological alterations including infiltration by inflammatory cells, damage to the alveolar epithelium and capillary endothelium, compromised alveolar epithelial barrier integrity, and increased alveolar-capillary membrane permeability. These pathophysiological changes result in severe hypoxemia, respiratory distress, and reduced lung compliance as primary clinical features [13]. The causes of neonatal ARDS are multifaceted, ranging from intrapulmonary to extrapulmonary factors such as asphyxia, acidosis, infections, meconium inhalation, and lung injury due to mechanical ventilation [13].

### Statistical method

The normality of all measurement data was verified. Data conforming to a normal distribution were analyzed using the t-test, while those not adhering to this distribution were evaluated using a non-parametric approach, specifically the Mann–Whitney U test. Categorical data were analyzed using Fisher’s exact test. Statistical analyses were performed using SPSS Statistics 26. The data in accordance with the normal distribution are represented by the mean ± standard deviation, and those that do not conform to the normal distribution are represented by the median [quartile interval]. With a *p*-value of less than 0.05 considered statistically significant.

## Result

This study included 18 neonates with respiratory and circulatory failure who were treated with ECMO support. The survival rate was 66.7% (12 out of 18). All 18 neonatal patients were successfully weaned off ECMO. A statistically significant difference was observed in the VIS and length of ICU stay between the survival and death groups (*P* < 0.05). However, there were no significant differences in gender, gestational age, weight, age at ECMO initiation, OI, duration of ECMO support, pulmonary artery pressure, creatinine clearance and liver function lesion, as shown in Table 1. The disease spectrum included ARDS, PPHN, MAS, severe pertussis infection (SPI) and CHD. The causes of death included cerebral hemorrhage (3 cases), and multiple organ failure (3 cases), as detailed in Table 2.

**Table 1:** Clinical characteristics of VA -ECMO neonates between the two groups

	**Survival group** **(*n* = 12)**	**Death group** **(*n* = 6)**	***P***
Male / female	8/4	5/1	0.615
Gestational age (weeks)	39.2[36.9–39.9]	38.2[32.7–40.2]	0.639
Age at ECMO (d)	2.0[1.0–2.8]	2.0[1.0–7.8]	0.842
Weight (kg)	3.5 ± 0.4	3.0 ± 1.0	0.218
OI	53.5 ± 15.3	75.0 ± 51.1	0.356
VIS	54.0[50.0–58.8]	62.5[56.5–86.0]	0.014
ECMO duration (h)	84.0[76.0–88.3]	63.5[47.3–150.5]	0.260
ICU (d)	24.0 ± 14.0	10.0 ± 5.3	0.032
Creatinine clearance^a^	22.3 ± 9.1	24.8 ± 9.4	0.590
Liver function lesion^a^	2	3	0.176
Pulmonary artery pressure^a^	69.5 ± 13.9	74.2 ± 21.8	0.586

*V-A ECMO* Veno-arterial extracorporeal membrane oxygenation, *OI* oxygenation index, *VIS* vasoactive inotropic score, *ICU* intensive care units

^a^Expressed as prior to ECMO treatment

**Table 2:** Disease distribution of the two groups

	**Survival group**	**Death group**	**Death reason**
PPHN	1	2	cerebral hemorrhage
PPHN + ARDS	6	1	multiple organ failure
MAS + ARDS	2	1	cerebral hemorrhage
CDH + PPHN	2	1	multiple organ failure
SPI + PPHN	1	1	multiple organ failure

*PPHN* Persistent pulmonary hypertension of the newborn, *ARDS* Acute respiratory distress syndrome, *MAS* Meconium aspiration syndrome, *CDH* Congenital diaphragmatic hernia, *SPI* Severe pertussis infection

**Fig. 1:**
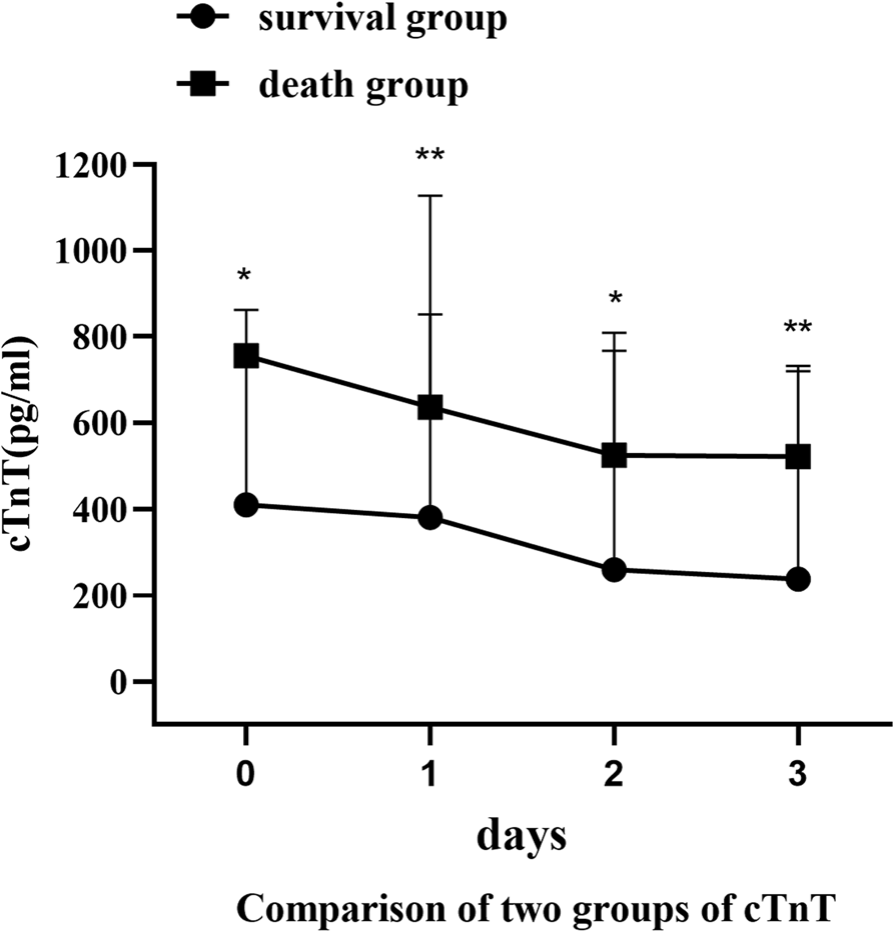
The trend chart of cTnT before and after ECMO treatment

**Fig. 2:**
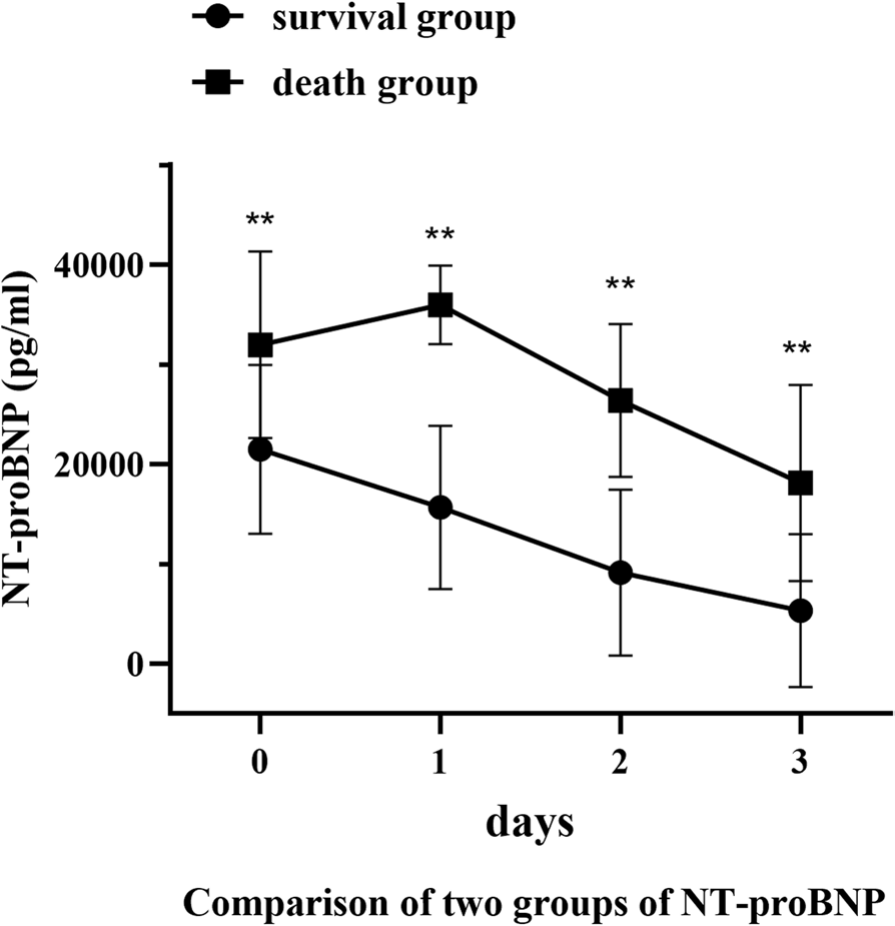
The trend chart of NT-proBNP before and after ECMO treatment

Tables 3 and 4, Figs. 1 and 2 illustrate the changes in serum cTnT and NT-proBNP levels in the two groups. The absolute values of serum cTnT and NT-proBNP were significantly higher in the death group compared to the survival group, with notable statistical differences observed on the day of ECMO initiation, and at 24, 48, and 72 h post-ECMO treatment (*P* < 0.05). Serum cTnT and NT-proBNP levels in both groups showed a gradual decrease as ECMO duration increased. Furthermore, the rate of decline was significantly greater in the survival group, with significant statistical differences (*P <* 0.05). In both groups, the most substantial decrease in cTnT occurred on the first day of post-ECMO treatment, whereas NT-proBNP showed the most significant decline on the second day of post-ECMO support. Notably, in this study, both cTnT and NT-proBNP levels did not show an early decrease in the death group.

**Table 3:** Comparison of two groups of cTnT

	**Survival group**	**Death group**	***P***
cTnT0	286[182–529]	754[670–848]	0.007
cTnT1	121[91–285]	583[485–747]	0.004
cTnT2	84[74–175]	473[208–873]	0.005
cTnT3	754[670–848]	462[358–658]	0.002
ΔcTnT1-0	0.50[0.41–0.58]	0.22[0.11–0.28]	0.004
ΔcTnT2-0	0.61 ± 0.16	0.31 ± 0.33	0.026
ΔcTnT3-0	0.73[0.65–0.84]	0.42[-0.03–0.53]	0.007

**Table 4:** Comparison of two groups of NT-proBNP

	**Survival group**	**Death group**	***P***
NT-proBNP0	21,838 ± 8457	35,288 ± 4652	0.002
NT-proBNP1	15,831 ± 8256	35,983 ± 4322	0.000
NT-proBNP2	5227 ± 3634	30,208 ± 4260	0.000
NT-proBNP3	3656 ± 3131	18,129 ± 10,787	0.021
ΔNT-proBNP1-0	0.29 ± 0.23	-0.04 ± 0.20	0.011
ΔNT-proBNP2-0	0.77 ± 0.14	0.13 ± 0.18	0.000
ΔNT-proBNP3-0	0.84 ± 0.11	0.48 ± 0.32	0.037

## Discussion

This retrospective study analyzed early serum cTnT and NT-proBNP levels in neonates weighing less than 4 kg and within one week of age, requiring ECMO support for respiratory and circulatory failure. The findings revealed that early absolute values of cTnT and NT-proBNP in the death group were significantly higher than those in survivors, with a notably lower rate of decline. ECMO technology facilitates circulation and gas exchange, providing time for the recovery of compromised respiratory and circulatory systems [14, 15]. While many studies have explored cTnT and NT-proBNP in ECMO treatment of fulminant myocarditis, research focusing on neonates under 4 kg and receiving ECMO within their first week is limited. Additionally, clinical evaluations of cTnT and NT-proBNP are predominantly based on adult data, with scant reports on these biomarkers in low-weight newborns post-ECMO initiation. Therefore, this study underscores the importance of further research on ECMO treatment in low-weight neonates, an area requiring more focused attention.

Various studies have established that myocardial cell ischemia and hypoxia, arising from multiple causes, can result in elevated serum cTnT levels [16, 17]. Conditions like PPHN and ARDS can lead to respiratory and circulatory failure, causing diffuse and progressive myocardial damage, often evidenced by a sustained early increase in cTnT. ECMO therapy, which involves using a centrifugal pump to draw blood from the vena cava, can decrease cardiac preload and left ventricular end-diastolic volume, thereby reducing cardiac oxygen consumption. It also increases blood pressure, ensures cardiac oxygen supply, and prevents myocardial damage due to hypoperfusion, enabling an earlier decrease in cTnT levels [18]. These findings align with the results observed in our study.

In our cases, there was a significant decrease in serum cTnT following ECMO treatment. Interestingly, we observed an increase in cTnT on the second day post-ECMO initiation, associated with mortality, suggesting that a lack of decrease in cTnT after ECMO may indicate a poor prognosis. Additionally, a notable increase in serum cTnT was observed after ECMO weaning, possibly due to increased cardiac workload and oxygen consumption, warranting further investigation. Hence, beyond the absolute value of serum cTnT, the decline rate warrants closer attention and might serve as a reliable predictor of neonatal ECMO outcomes. Considering cTnT as a macromolecule, its clearance is influenced by factors like protein levels and liver function [19]. During our ECMO treatments, albumin was routinely administered, maintaining high protein levels in each neonate. Liver function was impaired in 5 patients prior to ECMO, but the difference between the groups was not statistically significant.

Thus, we infer that protein levels and liver function minimally impacted the overall troponin changes, making troponin clearance comparisons informative. The difference in VIS between the two groups of neonates before ECMO treatment was statistically significant, and we concluded that there was a greater circulatory dysfunction in the neonates in the death group; and its prognostic value for the neonates needs to be further investigated.

BNP is a polypeptide neurohormone primarily synthesized in the ventricles. Initially, ventricular myocytes produce a physiologically inactive hormone precursor, which is then cleaved into BNP and NT-proBNP by endonucleases. Increased ventricular volume load elevates wall tension and can impair systolic or diastolic ventricular function, leading to the synthesis and release of NT-proBNP, and subsequently, higher NT-proBNP levels [20, 21]. Neonatal respiratory and circulatory failure, characterized by reduced cardiac contractility and cardiac blood stasis, results in increased wall tension and a consequent rise in NT-proBNP. ECMO support reduces cardiac workload, oxygen consumption, and mechanical shear force, which may contribute to the observed decrease in NT-proBNP levels during ECMO [22]. NT-proBNP is primarily eliminated by the kidneys, and we assessed the creatinine clearance of each neonate prior to ECMO treatment. Our findings indicated comparable creatinine clearance levels between the two neonatal groups, suggesting equivalent renal function. Additionally, during ECMO, full blood flow is achieved in the VA-ECMO mode, ensuring adequate renal perfusion and minimizing the effects of renal insufficiency or poor renal perfusion on NT-proBNP metabolism.

In this study, NT-proBNP levels significantly decreased under ECMO support. Falkensammer et al. noted that BNP levels could reflect left ventricular stretch, serving as a valuable marker for monitoring left ventricular dilation [23]. Reynolds et al. posited that BNP levels correlate with right heart function, and in children with PPHN, BNP could be a prognostic indicator [24]. Baptista and colleagues, in their evaluation of NT-proBNP in 13 neonates with CDH, found that elevated levels were predictive of mortality [25]. Our findings align with these studies, showing that NT-proBNP levels in the death group were significantly higher than those in the survival group, with a notably slower decline rate. Specifically, patients who did not exhibit a decrease in NT-proBNP within 24 h post-ECMO initiation were all in the death group, suggesting that stable or increasing NT-proBNP levels post-ECMO initiation may be indicative of a poor prognosis. These results underscore the importance of monitoring not only the absolute levels of cTnT and BNP but also their dynamic changes, which may offer more effective prognostic insights than absolute values alone.

## Limitation

The study faced certain limitations. The primary limitation was the small sample size. Our focus on underweight neonates receiving ECMO treatment within their first week of life limited the pool of eligible participants, as the incidence of neonates requiring ECMO in this specific subgroup is relatively low. Additionally, the small sample size hindered the ability to conduct an effective statistical analysis of prognostic factors. Furthermore, this study was a single-center retrospective analysis. In the future, we plan to collaborate in a multi-center study to more comprehensively analyze factors influencing the prognosis of underweight newborns receiving ECMO treatment within one week of birth.

## Conclusion

The variations in both the absolute values and decline rates of early serum cTnT and NT-proBNP in neonates undergoing V-A ECMO may serve as predictors of their prognosis. However, this potential correlation necessitates further investigation.

## Data Availability

The data that support the findings of this study are available on request from the corresponding author. The data are not publicly available due to privacy or ethical restrictions.

## References

[1] Kattan J, González A, Becker P, Faunes M, Estay A, Toso P, Urzúa S, Castillo A, Fabres J (2013). Survival of newborn infants with severe respiratory failure before and after establishing an extracorporeal membrane oxygenation program. Pediatr Crit Care Med.

[2] Bartlett RH, Gattinoni L (2010). Current status of extracorporeal life support (ECMO) for cardiopulmonary failure. Minerva Anestesiol.

[3] Gualandro DM, Puelacher C, Mueller C (2014). High-sensitivity cardiac troponin in acute conditions. Curr Opin Crit Care.

[4] Dentali F, Cei M, Mumoli N, Gianni M (2015). How to predict short- and long-term mortality in patients with pulmonary embolism?. Pol Arch Med Wewn.

[5] Said F, Haarman MG, Roofthooft MTR, Hillege HL, Ploegstra MJ, Berger RMF (2020). Serial measurements of N-terminal pro-B-type natriuretic peptide serum level for monitoring pulmonary arterial hypertension in children. J Pediatr.

[6] Jenks CL, Raman L, Dalton HJ (2017). Pediatric extracorporeal membrane oxygenation. Crit Care Clin.

[7] Yamazaki Y, Oba K, Matsui Y, Morimoto Y (2018). Vasoactive-inotropic score as a predictor of morbidity and mortality in adults after cardiac surgery with cardiopulmonary bypass. J Anesth.

[8] Wild KT, Rintoul N, Kattan J, Gray B (2020). Extracorporeal Life Support Organization (ELSO): guidelines for neonatal respiratory failure. ASAIO J.

[9] Bhattacharya S, Sen S, Levy PT, Rios DR (2019). Comprehensive evaluation of right heart performance and pulmonary hemodynamics in neonatal pulmonary hypertension : evaluation of cardiopulmonary performance in neonatal pulmonary hypertension. Curr Treat Options Cardiovasc Med.

[10] Lv GJ, Li AL, Tao XC, Zhai YN, Zhang Y, Lei JP, Gao Q, Xie WM, Zhai ZG (2022). The accuracy and influencing factors of Doppler echocardiography in estimating pulmonary artery systolic pressure: comparison with right heart catheterization: a retrospective cross-sectional study. BMC Med Imaging.

[11] Kovacs G, Dumitrescu D, Barner A, Greiner S, Grünig E, Hager A, Köhler T, Kozlik-Feldmann R, Kruck I, Lammers AE, Mereles D, Meyer A, Meyer J, Pabst S, Seyfarth HJ, Sinning C, Sorichter S, Stähler G, Wilkens H, Held M (2018). Definition, clinical classification and initial diagnosis of pulmonary hypertension: updated recommendations from the Cologne Consensus Conference 2018. Int J Cardiol.

[12] Singh Y, Lakshminrusimha S (2021). Pathophysiology and management of persistent pulmonary hypertension of the newborn. Clin Perinatol.

[13] Chi M, Mei YB, Feng ZC (2018). A review on neonatal acute respiratory distress syndrome. Zhongguo Dang Dai Er Ke Za Zhi.

[14] Chong SZ, Fang CY, Fang HY, Chen HC, Chen CJ, Yang CH, Hang CL, Yip HK, Wu CJ, Lee WC (2018). Associations with the In-Hospital Survival Following Extracorporeal Membrane Oxygenation in Adult Acute Fulminant Myocarditis. J Clin Med.

[15] Astoria MT, Karam SE, Moores RR, Rozycki HJ (2015). Cardiac Troponin Levels in Neonates Who Require ECMO for Noncardiac Indications Are Elevated in Nonsurvivors. Am J Perinatol.

[16] Dursunoğlu N, Dursunoğlu D, Yıldız Aİ, Rota S (2016). Evaluation of cardiac biomarkers and right ventricular dysfunction in patients with acute pulmonary embolism. Anatol J Cardiol.

[17] Butto A, Rossano JW, Nandi D, Ravishankar C, Lin KY, O'Connor MJ, Shaddy RE, Shamszad P (2018). Elevated Troponin in the First 72 h of Hospitalization for Pediatric Viral Myocarditis is Associated with ECMO: An Analysis of the PHIS+ Database. Pediatr Cardiol.

[18] Zhu GJ, Sun LN, Li XH, Wang NF, Wu HH, Yuan CX, Li QQ, Xu P, Ren YQ, Mao BG (2015). Myocardial protection of early extracorporeal membrane oxygenation (ECMO) support for acute myocardial infarction with cardiogenic shock in pigs. Heart Vessels.

[19] Lazo M, Rubin J, Clark JM, Coresh J, Schneider AL, Ndumele C, Hoogeveen RC, Ballantyne CM, Selvin E (2015). The association of liver enzymes with biomarkers of subclinical myocardial damage and structural heart disease. J Hepatol.

[20] Krishnan B, Patarroyo-Aponte M, Duprez D, Pritzker M, Missov E, Benditt DG (2015). Orthostatic hypotension of unknown cause: Unanticipated association with elevated circulating N-terminal brain natriuretic peptide (NT-proBNP). Heart Rhythm.

[21] Nagaya N, Nishikimi T, Okano Y, Uematsu M, Satoh T, Kyotani S, Kuribayashi S, Hamada S, Kakishita M, Nakanishi N, Takamiya M, Kunieda T, Matsuo H, Kangawa K (1998). Plasma brain natriuretic peptide levels increase in proportion to the extent of right ventricular dysfunction in pulmonary hypertension. J Am Coll Cardiol.

[22] Takase H, Dohi Y (2014). Kidney function crucially affects B-type natriuretic peptide (BNP), N-terminal proBNP and their relationship. Eur J Clin Invest.

[23] Falkensammer CB, Heinle JS, Chang AC (2008). Serial plasma BNP levels in assessing inadequate left ventricular decompression on ECMO. Pediatr Cardiol.

[24] Reynolds EW, Ellington JG, Vranicar M, Bada HS (2004). Brain-type natriuretic peptide in the diagnosis and management of persistent pulmonary hypertension of the newborn. Pediatrics.

[25] Baptista MJ, Rocha G, Clemente F, Azevedo LF, Tibboel D, Leite-Moreira AF, Guimarães H, Areias JC, Correia-Pinto J (2008). N-terminal-pro-B type natriuretic peptide as a useful tool to evaluate pulmonary hypertension and cardiac function in CDH infants. Neonatology.

